# Exploring the Caste-Specific Multi-Layer Defense Mechanism of Formosan Subterranean Termites, *Coptotermes formosanus* Shiraki

**DOI:** 10.3390/ijms18122694

**Published:** 2017-12-12

**Authors:** Abid Hussain, Ming-Yi Tian, Shuo-Yang Wen

**Affiliations:** 1 Department of Entomology, College of Natural Resources and Environment, South China Agricultural University, Guangzhou 510640, China; abhussain@kfu.edu.sa (A.H.); mytian@scau.edu.cn (M.-Y.T.); 2 Laboratory of Bio-control and Molecular Biology, Department of Arid Land Agriculture, College of Agricultural and Food Sciences, King Faisal University, 31982 Hofuf, Al-Ahsa, Saudi Arabia

**Keywords:** antioxidant defense, castes, disease resistance, HS-SPME, SEM, termites, volatiles

## Abstract

The survival and foraging of *Coptotermes formosanus* Shiraki in a microbe-rich environment reflect the adaptation of an extraordinary, sophisticated defense mechanism by the nest-mates. We aimed to explore the host pathogen interaction by studying caste-specific volatile chemistry and genes encoding the antioxidant defense of winged imagoes, nymphs, soldiers and workers of Formosan subterranean termites. Qualitative analyses of *C. formosanus* Shiraki performed by HS-SPME/GC-MS showed considerable variations in the chemical composition of volatile organic compounds (VOCs) and their proportions among all the castes. Winged imagoes produced the most important compounds such as naphthalene and *n-*hexanoic acid. The antifungal activity of these compounds along with nonanal, *n*-pentadecane, *n-*tetradecane, *n*-heptadecane and methyl octanoate against the conidial suspensions of *Metarhizium anisopliae* and *Beauveria bassiana* isolates enable us to suggest that the failure of natural fungal infection in the nest is due to the antiseptic environment of the nest, which is mainly controlled by the VOCs of nest-mates. In addition, conidial germination of *M. anisopliae* and *B. bassiana* isolates evaluated on the cuticle of each caste showed significant variations among isolates and different castes. Our results showed that the conidia of *M. anisopliae* 02049 exhibited the highest germination on the cuticle of all the inoculated castes. Moreover, we recorded the lowest germination of the conidia of *B. bassiana* 200436. Caste-specific germination variations enabled us to report for the first time that the cuticle of winged imagoes was found to be the most resistant cuticle. The analysis of the transcriptome of *C. formosanus* Shiraki revealed the identification of 17 genes directly involved in antioxidant defense. Expression patterns of the identified antioxidant genes by quantitative real-time PCR (qPCR) revealed the significantly highest upregulation of *CAT*, *GST*, *PRXSL*, *Cu/Zn-SOD2*, *TXN1*, *TXN2*, *TXNL1*, *TXNL2*, *TXNL4A* and *TPx* genes among winged imagoes upon infection with the most virulent isolate, *M. anisopliae* 02049. Furthermore, soldiers showed the least expression of genes encoding antioxidant defense. Our findings indicated that the volatile chemistry of nest-mates and genes encoding antioxidant defense greatly contribute to the survival and foraging of Formosan subterranean termites in a microbe-rich habitat.

## 1. Introduction

Formosan subterranean termites, *Coptotermes formosanus* Shiraki (Isoptera: Rhinotermitidae), a possible threat to the economies of the world, live in complex networks of galleries or tunnels below the soil surface or encased in wood in close social groups consisting of millions of individuals jammed together within the nest. Wood and other plant tissues are the main sources of cellulose, which is the principal component of the termite’s diet. Therefore, termites are considered ecologically important because they facilitate the cellulose degradation that ultimately recycles the nutrients back into the soil [1]. However, their beneficial role as a decomposer changes to “pest” when they encounter commodities important for human consumption including forest and agronomic vegetation used for construction and human consumption, respectively. The huge economic losses caused by the termites all around the world have over-shadowed their beneficial role as decomposers. Previously, a great variety of termiticides were tried due to their cryptic mode of existence [2,3]. However, concerns over human health and environmental pollution by liquid termiticides and the limitations of both the non-repellant and repellent termiticides have provided the impetus to look for alternative methods to control termites.

Naturally-occurring bio-control agents, especially entomopathogenic fungi, are important alternatives in reversing termite management’s dependence on hazardous liquid termiticides. Termite metabolic activity and social behavioral interactions of the nest mates create a conducive nest environment for the development and auto-transmission of soil-borne entomopathogenic fungi dwelling in their surroundings [4,5]. In spite of numerous successful laboratory studies of virulent isolates of entomopathogenic fungi against termites, the colonies of termites are rarely reported to be destroyed by fungal infections. Their failure prompted scientists to investigate the disease resistance mechanism. Recent developments made in this field suggested that *C. formosanus* Shiraki have adapted a number of defense mechanisms such as behavioral adaptations [4,5,6], immune responses [7,8] and chemical defenses [9,10] to limit the spread of fungal inoculums among the nest-mates. In this regard, *C. formosanus* Shiraki antioxidant defense genes that respond to reactive oxygen species (ROS) generated after fungal infections were neglected. ROS are produced naturally in the form of superoxide anions and hydroxyl radicals as a consequence of oxidative metabolism. Nature has created an intrinsic balance between ROS and antioxidant processes. However, stressful situations significantly enhance the levels of oxidative damage in the target host that ultimately lead to oxidative stress [11,12]. It is of particular interest to dissect the molecular basis of *C. formosanus* Shiraki antioxidant defense as termites thrive in an environment thought to offer conditions highly favorable for sustaining fungal infection.

Formosan subterranean termites have in part adapted to the disease risk posed by microbes by employing chemicals capable of suppressing fungal pathogens and competitors [13,14]. These substances permanently flow through the close environments of the nest, which maintain not only the integration of the colony, but also provide defense [9,15]. In addition, it has also been reported that *C. formosanus* nests contain naphthalene, which shows fungistatic activity [9]. The volatility of naphthalene qualifies it to permeate into the complex network of galleries of termite nests as a chemical defense against natural pathogens. However, their findings did not determine the source of this substance. Later on, they suggested that naphthalene might be produced as a byproduct of cellulose digestion by the termites’ microflora [16].

In the past, several investigations have been carried out in order to identify the hydrocarbons of termite cuticle, nest and feces through surface hexane extraction procedures [9,17,18]. However, there are few reports on the analysis of volatile organic compounds (VOCs) released by termites by SPME/GC-MS, which is a solvent-free form of analysis that provides additional beneficial information from live termites in a more natural state [19]. The range of VOCs produced by *C. formosanus* Shiraki from different castes has not been fully investigated. Our study aimed to (1) qualitatively analyze the VOCs of nymphs, winged imagoes, soldiers and workers of termites by HS-SPME using GC-MS, (2) test the antifungal response of major identified fractions of Formosan subterranean termites against isolates of *M. anisopliae* and *B. bassiana* known to colonize termites, (3) determine the percent viability of *M. anisopliae* and *B. bassiana* conidia on the cuticle of each tested caste (nymphs, winged imagoes, soldiers and workers), (4) analyze the expressed sequence tags (ESTs) to compile the antioxidant defense-related genes of *C. formosanus* Shiraki and (5) validate and quantify the expression pattern of each identified antioxidant gene in each tested caste against entomopathogenic fungal infections to unfold for the first time chemical and antioxidant disease resistance mechanism of Formosan subterranean termites.

## 2. Results

### 2.1. Chemical Composition of Volatile Blends of Formosan Subterranean Termites

HS-SPME analyses of the volatiles have revealed an impressive diversity of chemical components in *C. formosanus* Shiraki (winged imagoes, nymphs, soldiers and workers): fatty acids, aldehydes, aromatics, heterocyclic aromatics, flavanoids, sesquiterpenes, straight-chained and branched alkanes, ketones, esters, alcohols and nitrogen- or sulfur-containing compounds (Table 1). Fourteen compounds ranging from C_6_–C_18_ hydrocarbons were identified from winged imagoes with naphthalene as the major component. Hence, volatiles from nymphs were comprised of C_10_–C_16_ hydrocarbons, with 1,2,3-trimethyl-4[E]-propenyl-naphthalene being the major component. The blend of soldier volatiles was comprised of C_9_–C_18_ hydrocarbons. Among the seventeen compounds, oleic acid was the major component. The volatile blend of workers was comprised of fifteen compounds ranging from C_9_–C_18_ hydrocarbons with *n*-amyl isovalerate being the major component (Table 1).

### 2.2. Antifungal Activity of Selected Compounds against Entomopathogenic Fungi

**Figure 1:**
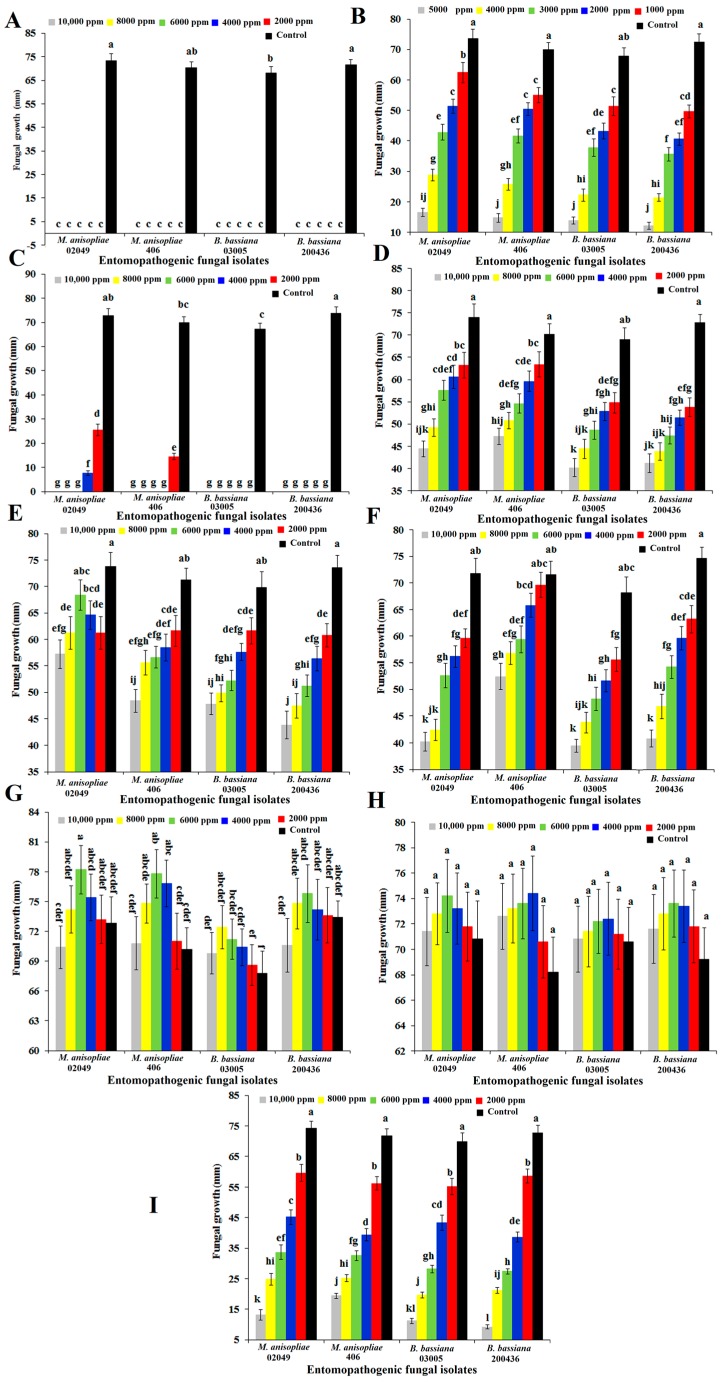
The effect of different concentrations of *n*-hexanoic acid (**A**), naphthalene (**B**), nonanal (**C**), *n*-pentadecane (**D**), *n*-tetradecane (**E**), *n*-heptadecane (**F**), *n*-hexadecane (**G**), oleic acid (**H**) and methyl octanoate (**I**) on the radial growth (12-d colony diameter) of entomopathogenic fungal isolates at 26 ± 1 °C, in complete darkness. Mean ± SE values followed by different letter(s) are significantly different (Fisher’s LSD test, α = 0.05). Values are the means of five replicates, and each replicate is the average of five Petri dishes.

Isolates of entomopathogenic fungi, viz. *M. anisopliae* and *B. bassiana*, were highly sensitive to nonanal and *n*-hexanoic acid, showing completely or nearly completely suppressed radial fungal growth at all tested doses (Figure 1A,C). Naphthalene at the highest dose (5000 ppm) showed less than 20-mm radial growth of tested isolates. However, naphthalene at all tested doses (*F* = 355.10; degree of freedom = 5, 96; *p* < 0.0001) against all tested isolates (*F* = 14.28; df = 3, 96; *p* < 0.0001) showed significant differences in their radial growth (Figure 1B). The mycelial growth of straight chain alkanes such as *n*-pentadecane (Figure 1D) and *n*-heptadecane (Figure 1F) comparatively showed a lower response even at the highest dose (10,000 ppm). Overall, the inversely proportional relationship of radial growth in response to different doses of *n*-pentadecane and *n*-heptadecane was observed. However, there were significant differences in radial growth at different doses of *n*-pentadecane (*F* = 78.32; df = 5, 96; *p* < 0.001), *n*-heptadecane (*F* = 87.62; df = 5, 96; *p* < 0.001) and *n*-tetradecane (*F* = 40.53; df = 5, 96; *p* < 0.001). Similarly, *n*-pentadecane (*F* = 16.34; df = 3, 96; *p* < 0.001), *n*-heptadecane (*F* = 30.92; df = 3, 96; *p* < 0.001) and *n*-tetradecane (*F* = 16.36; df = 3, 96; *p* < 0.001) also showed significant differences in the radial growth of all the tested isolates of entomopathogenic fungi (Figure 1D–F). The incorporation of *n*-hexadecane (Figure 1G) and oleic acid (Figure 1H) into the growth media (PDA) imparted enhanced radial growth impacts on all the tested isolates. However, *n*-hexadecane between 4000 and 6000 ppm and oleic acid between 6000 and 8000 ppm showed a growth stimulatory response against all tested isolates. However, the interaction effect of oleic acid (*F* = 0.09; df = 15, 96; *p* > 0.05) and *n*-hexadecane (*F* = 0.35; df = 15, 96; *p* > 0.05) at different doses against different isolates showed a non-significant interaction. Methyl octanoate greatly inhibited the radial growth of each tested isolate in a dose-dependent manner (Figure 1I). Radial growth in the presence of methyl octanoate differed significantly among all tested isolates (*F* = 9.71; df = 3, 96; *p* < 0.0001), doses (*F* = 557.46; df = 5, 96; *p* < 0.0001) and their interaction (*F* = 3.04; df = 15, 96; *p* < 0.0001).

### 2.3. Conidial Percent Germination of Entomopathogenic Fungi on the Cuticle of Different Castes of FSTs

**Figure 2:**
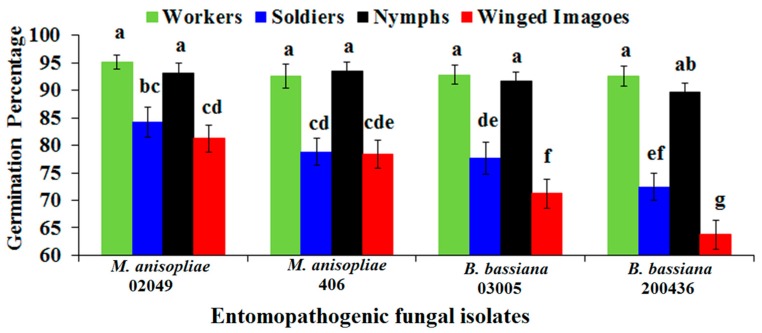
Percent germination of the conidia of entomopathogenic fungal isolates on the cuticle of workers, soldiers, nymphs and winged imagoes. Values are the means of five replicates. Mean ± SE values followed by different letter(s) are significantly different (Fisher’s LSD test, α = 0.05).

**Figure 3:**
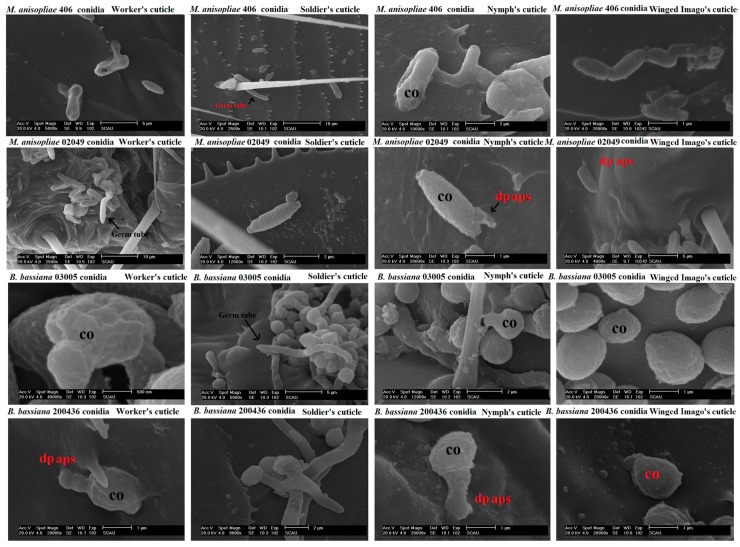
SEM used for the detection of growth patterns of tested isolates of entomopathogenic fungi on the cuticle of workers, soldiers, nymphs and winged imagoes of *C. formosanus* Shiraki. dp aps stands for directly-penetrating appressorium-like structure; co stands for unipolar-germinated conidium.

Conidial germination (%) significantly varied among the tested isolates of *M. anisopliae* and *B. bassiana* (*F* = 11.42; df = 3, 64; *p* < 0.0001), the cuticle of different castes (*F* = 77.69; df = 3, 64; *p* < 0.0001) and their interaction (*F* = 2.17; df = 9, 64; *p* < 0.05). Isolate of *M. anisopliae* 02049 exhibited the highest percent germination on the cuticle of all the inoculated castes compared with other isolates. However, *B. bassiana* 200436 exhibited significantly the lowest percent germination on the cuticle of each tested caste (Figure 2). The developmental events of each tested isolate on the cuticles of workers, soldiers, nymphs and winged imagoes are shown in Figure 3.

Overall, the cuticle of workers was found to be the most susceptible resulting in the highest percent germination of each tested isolate. However, percent germination of all isolates remained statistically on par with nymphs’ cuticle except isolate *B. bassiana* 200436. Moreover, the cuticle of winged imagoes was found to be the most resistant cuticle resulting in the lowest conidial percent germination of all tested isolates of entomopathogenic fungi (Figure 2).

### 2.4. Sequence Annotations for Antioxidant Genes of FSTs

In order to compile an antioxidant defense-related database for Formosan subterranean termites, ESTs were mined resulting in 17 clusters involved in antioxidant defense (Table 2). The clusters of identified antioxidant genes were directly involved in antioxidant defense of *C. formosanus* Shiraki (Table 2). Overall, antioxidant-related sequences matched with all important antioxidant gene classes such as *catalase*, *glutaredoxin*, *glutathione S-transferase*, *peroxiredoxin*, *superoxide dismutase Cu/Zn, superoxide dismutase Fe* (*Fe-SOD*), *thioredoxin family protein*, *thioredoxin peroxidase* and *thioredoxin-like [2Fe–2S] ferredoxin (Fd) family protein* (Table 2). Identified sequences directly involved in antioxidant defense were deposited to NCBI (GenBank Accession Numbers JX311467, JX876646, JX879124–JX879125, JX915904–JX915909, KC571990, KC632518, KC632520, KC741174–KC741177).

### 2.5. Quantitative Expression Patterns of FSTs Antioxidant Genes

The expressions of *CAT*, *DUOX1*, *GRX*, *GST*, *PRXS*, *PRXSL*, *PRXS1L*, *Cu/Zn-SOD1*, *Fe-SOD*, *Cu/Zn-SOD2*, *TXN1*, *TXN2*, *TXNL1*, *TXNL2*, *TXNL4A*, *TPx* and *TRx-like-Fd* determined in winged imagoes, nymphs, soldiers and workers upon infection with two isolates of *B. bassiana* and two isolates of *M. anisopliae* showed caste- and causal agent-specific expression patterns (Table 3). Among all the analyzed genes, *CAT* showed the highest transcript level. Interestingly, low expression (less than two-fold) of *DUOX1*, *PRXS1L* and *TXNL1* was observed upon fungal infection among winged imagoes, nymphs, soldiers and workers. *CAT*, *GST*, *PRXSL*, *Cu/Zn-SOD2*, *TXN1*, *TXN2*, *TXNL1*, *TXNL2*, *TXNL4A* and *TPx* transcript levels were found to be high among winged imagoes infected with *M. anisopliae* 02049. *PRXS*, *Fe-SOD* and *TRx-like-Fd* showed the highest expression among workers infected with fungal suspensions. Genes encoding dual oxidase 1, glutaredoxin like protein, superoxide dismutase Cu/Zn1 and superoxide dismutase Fe were highly expressed exclusively in nymphs of *C. formosanus* Shiraki. Overall, soldiers displayed low expression levels of tested antioxidant genes compared with nymphs, workers and winged imagoes. Furthermore, our results suggested that *M. anisopliae* 02049 greatly enhanced the expression of all antioxidant genes tested, whereas *B. bassiana* 200436 failed to induce the expression of antioxidant genes.

## 3. Discussion

The successful foraging and amazing social lives of Formosan subterranean termites in a microbe-rich environment are the manifestation of an extraordinary, sophisticated defense mechanism of the colony members. The identification and origin of important compounds from VOCs of colony members with proven antifungal activity and mining the genes encoding the antioxidant defense of *C. formosanus* Shiraki and their expression patterns against fungal infections revealed in the current study profoundly widen the knowledge on disease resistance in termites in addition to previously explored social behavioral disease resistance interactions [4,5,20,21] and the immune mechanisms of disease resistance [7].

Host cuticle is the first line of defense against invading fungal pathogens [22]. Conidial germination patterns recorded on the cuticle of winged imagoes, nymphs, soldiers and workers in the current study demonstrated that the percent of germination significantly changed with fungal agent and colony members (Figure 2). The highest estimates of percent germination of all tested isolates on the cuticle of workers indicate their suitability for infection. The least virulent isolate (*B. bassiana* 200436) reported before against *C. formosanus* Shiraki workers [4] showed highly significant variations in the current study among the cuticles of tested castes. A high percent of germination on the cuticle of workers and nymphs, while a significantly lesser percent of germination on the cuticle of soldiers and winged imagoes suggest exploring the chemistry of each caste profile in order to discover the chemical defense mechanism of *C. formosanus* Shiraki.

Volatile organic compound profiles of winged imagoes, nymphs, soldiers and workers analyzed by HS-SPME unveiled an impressive diversity of compounds. Furthermore, our results reflected remarkable differences in the production of each caste’s volatiles suggesting that the proportion and volatile profile of *C. formosanus* Shiraki vary among the castes (winged imagoes, nymphs, soldiers and workers). This interpretation is consistent with previous work demonstrating that variability exists among the damp-wood termites [23,24]. In contrast, the study of Haverty et al. [25] on inter-colony GC-MS analysis of *C. formosanus* cuticular hydrocarbon composition revealed little variations among colony members. The difference might be because of the methodology for the extraction of hydrocarbons. Trail-forming *n-*hexanoic acid and naphthalene for the first time were identified in association with winged imagoes. Previously, naphthalene was found to be associated with nest material [16] and was assumed to be produced as a result of the natural decomposition of wood or fumigation by nest-mates; while *n-*hexanoic acid was previously detected from the sternal gland of *Z. angusticollis* [26]. Furthermore, our results for the antifungal investigations of naphthalene against *M. anisopliae* and *B. bassiana* are comparable to those obtained in the earlier investigations of Wright et al. [10], who exposed *M*. *anisopliae* to several concentrations of naphthalene and fenchone. They observed significant growth inhibition at higher concentrations as observed in our study, enabling us to suggest that *M. anisopliae* and *B. bassiana* cultures are highly sensitive to naphthalene at higher doses. Our results confirm and extend those of Wiltz et al. [27], who used naphthalene, butylated hydroxytoluene, dioctyl phthalate and adipic dioctyl ester against saprophytic *Mucor* sp. and suggested that a high concentration of naphthalene inhibited the growth of *Mucor* sp. Our results suggested that a high proportion of naphthalene production by winged imagoes (20.02%) favored the evolution of biochemical protection against pathogens. Another important component, *n-*hexanoic acid (1.35% of total nymphs VOCs), is reported to have antifungal activity against entomopathogenic fungi [26]. Significant reduction in growth in the current research supports the idea that *n-*hexanoic acid had the original function of controlling microbes within the nest, and their prominent role in communication may have evolved secondarily. On the basis of the above findings, it may be speculated that the versatility of primary reproductive (PR) volatile compounds contributed to the success of incipient colonies.

To date, the inhibitory effect of the volatiles such as nonanal, methyl octanoate, *n*-tetradecane, *n*-pentadecane and *n*-heptadecane against entomopathogenic fungi have not yet been investigated. Complete inhibition of entomopathogenic fungi was observed for nonanal suggesting that the presence of nonanal in the VOC profile of soldiers imparts strong fungistatic activity to the entomopathogenic fungi. In addition, our results suggest that *n*-hexadecane and oleic acid stimulated the growth of fungal isolates. Stimulation of mycelial growth by *n*-hexadecane and oleic acid is a novel finding. In addition, previous studies on the cue synergism in termites have reported repellent properties of oleic acid [28]. In contrast, oleic acid was found to be antifungal by significantly reducing the mycelial growth of the plant pathogenic fungus, *Pythium ultimum* [29]. The differences in biological activity of VOC from different castes suggest the possibility that different castes are capable of emitting specific biologically-active compounds pertinent to their roles in the life of the colony, which is intriguing and important. Normally, it is assumed that the task of defense is performed only by the soldier caste, which is morphologically specialized for this purpose [30], and the workers are involved in nest construction, feeding (trophallaxis) and cleaning (allogrooming). However, the discovery of hazardous compounds (naphthalene and *n*-hexanoic acid) from the winged imagoes, which may act as a fumigant, reflected the possible function of the production of VOCs by the PRs in a controlled microenvironment having no direct air exchange, which made fumigation a possible defense, not only against pathogens, but also predators.

Fungal infection imparted oxidative stress in the target host by generating reactive oxygen species (ROS). In order to return the cells to homeostasis, efficient elimination of ROS to avoid lipids’, proteins’ and nucleic acids’ damage, insects have evolved an important, complex antioxidant system [12]. The transcriptomic analysis of Formosan subterranean termites revealed the identification of numerous genes involved in antioxidant defense. The identified antioxidant genes (*CAT*, *DUOX1*, *GRXL*, *GST*, *PRXS*, *PRXSL*, *PRXS1L*, *Cu/Zn-SOD1*, *Fe-SOD*, *Cu/Zn-SOD2*, *TXN1*, *TXN2*, *TXNL1*, *TXNL2*, *TXNL4A*, *TPx* and *TRx-like-Fd*) showed homology with similar genes from other species. All the identified antioxidant genes have direct antioxidant effects. However, *transferrin* [31], *ferritin* [32] and *vitellogenin* [33], identified from the Formosan subterranean termites, were not included as they mediate indirect antioxidant effects. The molecularly-driven identification of well-developed antioxidant defense for the first time in *C. formosanus* Shiraki is a corroboration of their adaptation to a microbe-rich environment.

Infection of workers, soldiers, nymphs and winged imagoes of *C. formosanus* Shiraki with the conidial suspension of *B. bassiana* and *M. anisopliae* induced the generation of reactive metabolites such as O_2_^●−^ (superoxide anion) and H_2_O_2_ (hydrogen peroxide). In the current study, we identified three different first-line antioxidant genes such as *Cu/Zn-SOD1*, *Cu/Zn-SOD2* and *Fe-SOD* that catalyze superoxide radicals (O_2_^●−^) into H_2_O_2_. Among them, *Cu/Zn-SOD2* showed the highest fold expression in winged imagoes against the most pathogenic isolate *M. anisopliae* 02049. The high upregulation is in agreement with other studies on the first-line insect antioxidant defense genes against fungal infection [34].

To protect biomolecules from oxidative damage by scavenging excessive ROS (Kodrík et al. 2015), multiple components of the thioredoxin system such as *TXN1*, *TXN2*, *TXNL1*, *TXNL2*, *TXNL4A*, *TPx* and *TRx-like-Fd* identified here are in line with a previous study suggesting their importance in redox-regulatory processes [35]. The expression of components of the thioredoxin system appears to show caste- and isolate-specific patterns: *TXNL4A* is highly expressed in winged imagoes; *TRx-like-Fd* exhibits higher expression in workers and winged imagoes against all fungal infections; *TXN1* and *TPx* mainly are expressed against isolate 02049 among workers and winged imagoes; *TXN2* is expressed at higher levels among nymphs and winged imagoes against the infection of isolate 02049 (Table 3), implying that thioredoxin components play a crucial role in antioxidant defense against virulent isolates in the mentioned castes. In the current study, another two components of the thioredoxin superfamily such as *glutathione-S-transferases* (*GSTs*) and *Glutaredoxin like protein* (*GRXL*) were also identified. The expression of *GST* in winged imagoes and *GRXL* in nymphs was found to be significantly stimulated by *M. anisopliae* 02049 infection, which is already reported as a virulent isolate due to low LT_50_ values [4,5,6].

*Catalase* (*CAT*) is an important ROS-scavenging antioxidant mainly involved in the removal of H_2_O_2_. *CAT* and components of peroxiredoxin (*PRXS*, *PRXSL* and *PRXS1L*) prevent oxidative damage by elimination of excessive H_2_O_2_ in many organisms [36,37]. The highest induction of *CAT* and *PRXS* among winged imagoes and workers against the most virulent isolate 02049 corroborated their role in maintaining redox homeostasis by protecting the host from toxic accumulation of ROS. *PRXS1L* contrastingly failed to be expressed against all the fungal infections. However, variations among different castes in response to different fungal infections contribute to the infection lethality of the invading pathogen.

## 4. Materials and Methods

### 4.1. Termite Collection and Maintenance

Nymphs, workers and soldiers of Formosan subterranean termites, *Coptotermes formosanus* Shiraki, were collected from Huolu Shan Forest Park, Guangzhou, China. Termites were maintained at 24–27 °C in complete darkness, in glass Petri dishes (115 mm × 20 mm) containing moist filter paper as food. Winged imagoes (alates) for bioassays were collected with an aspirator below street lights during swarms from Huolu Shan Forest Park, Guangzhou, China.

### 4.2. Extraction and Analysis of VOCs Released by the Termites

Volatile organic compounds were extracted by SPME, with a 1-cm coated fiber (75 µm d_f_) of carboxen/polydimethylsiloxane (CAR/PDMS) from Supelco. Winged imagoes, nymphs, soldiers and workers (100 each, alive) of *C. formosanus* Shiraki were placed in separate glass vials (20 mL). Termites were equilibrated for 30 min at room temperature prior to inserting in SPME fiber. After this time, SPME fiber was exposed to the head space of the sample (winged imagoes, nymphs, soldiers and workers) for 30 min. The fiber was then retracted and inserted immediately into the inlet of the GC-MS for thermal desorption. The fiber was previously conditioned according to the manufacturer’s instructions and systematically reconditioned before each analysis. Blank injections were obtained by exposing the SPME fiber to clean empty vials. Substances found in the blank were subtracted from the VOC profile of each caste.

The qualitative analysis of the SPME extract of each caste was performed by GC-MS (Finnigan TRACE, Thermo Electron Corporation, Austin, TX, USA). The GC-MS was equipped with the HP1 capillary column having a 1-μm film thickness and 30 m × 0.25 mm internal diameter. Helium gas with 99.99% purity at a constant flow rate of 1.0 mL/min was used as the carrier gas. After injecting each sample, the initial oven temperature (45 °C) was maintained for 1 min. Subsequently, the temperature was raised to 100 °C at a rate of 5 °C/min and held for 2 min, followed by a gradient to 180 °C at a rate of 10 °C/min and held for 10 min. Mass spectra from 35–335 atomic mass units (amu) were repetitively scanned. Electron ionization (EI) was induced at 70 eV. Preliminary identification of VOCs of each *C. formosanus* Shiraki caste was made through comparison with the spectra in the Wiley275.1 and NIST98 libraries. Peaks of important compounds were confirmed by comparing the retention time and mass spectra with pure standards purchased from different suppliers.

### 4.3. Origin and Maintenance of Fungal Cultures

Four isolates of entomopathogenic fungi were selected to carry out fungal viability on host cuticle, fungal radial growth inhibition on PDA and host antioxidant defense experimentation. Two isolates, each from *Metarhizium anisopliae* (EBCL 02049) and *Beauveria bassiana* (EBCL 03005) isolated from *C. formosanus*, and two isolates, each from *Metarhizium anisopliae* (406) and *Beauveria bassiana* (200436) isolated from soil, were selected for whole experimentation. Each culture was maintained on potato dextrose agar (PDA) at 26 ± 1 °C, in complete darkness as mentioned in previous studies [5,6].

### 4.4. Preparation of Fungal Conidial Suspensions

Conidial suspension of each tested isolate of entomopathogenic fungi was prepared in sterile distilled water with 0.05% Tween 80 (Sigma-Aldrich, St. Louis, MO, USA) by harvesting 24-day-old sporulating cultures. The conidial suspension (1.3 × 10^7^ conidia/mL) and viability (93–99%) were calculated as described previously [38,39].

### 4.5. Antifungal Assays

The antifungal response of *n-*hexanoic acid (International Laboratory IL, San Francisco, CA, USA), naphthalene (Sinopharm Chemical Reagent Company, Shanghai, China), nonanal (International Laboratory IL), *n-*tetradecane (Sigma-Aldrich), *n*-pentadecane (Sigma-Aldrich), *n*-hexadecane (Sigma-Aldrich), *n*-heptadecane (Sigma-Aldrich), oleic acid (Sigma-Aldrich) and methyl octanoate (Sigma-Aldrich) was determined by the poison food technique. In brief, a specific range of different doses including 2000, 4000, 6000, 8000 and 10,000 ppm by adding 20, 40, 60, 80 and 100 µL of each tested compound (nonanal, *n-*hexanoic acid, naphthalene, *n-*tetradecane, *n*-pentadecane, *n*-hexadecane, *n*-heptadecane, oleic acid and methyl octanoate) was mixed with 10 mL PDA after sterilization at 50 °C. In the case of naphthalene, 10, 20, 30, 40 and 50 mg were mixed with sterilized PDA at 50 °C. Ten milliliters of PDA were poured into each Petri dish, separately. A five-microliter conidial suspension (1.3 × 10^7^ conidia/mL) of each tested isolate such as *M. anisopliae* 02049, *M. anisopliae* 406, *B. bassiana* 03005 and *B. bassiana* 200436 was pipetted to the center of each Petri dish (90 millimeter × 15 mm), separately. The inoculated agar plate was closed and sealed with laboratory film (Parafilm^®^ Pechiney Plastic Packaging; Menasha. WI, USA) and incubated at 26 ± 1 °C, in complete darkness. The radial growth (average of five perpendicular radial lengths) was recorded 12 days post-inoculation. Each treatment was applied to five adjacent Petri dishes for each replicate. The experiment was replicated five times by using separate cultures of each tested isolate. Similarly, a control experiment was also run simultaneously using 0.05% Tween 80. Two factor factorial analysis consisting of four isolates of entomopathogenic fungi and six doses were conducted for radial growth. Significant differences among means were indicated by Fisher’s least significant difference test (α = 0.05) [40].

### 4.6. Conidial Germination on the Cuticle of Formosan Subterranean Termites

Each caste (nymphs, winged imagoes, soldiers and workers) was infected by immersing them into the conidial suspension (1.3 × 10^7^ conidia/mL) of *M. anisopliae* 02049, *M. anisopliae* 406, *B. bassiana* 03005 and *B. bassiana* 200436 separately for 5 s into micro-centrifuge tubes by gentle swirling [7]. Five replicates, each from separate insects, were prepared likewise. Germination of each isolate on each caste cuticle was calculated by counting 100 conidia after 18 and 36 h post-inoculation by compound microscope and scanning electron microscope. Percent of germination data on termite’s cuticles were analyzed by two-factor factorial analysis comprised of four castes and four isolates of entomopathogenic fungi. Significant differences among means were indicated by Fisher’s least significant difference test (α = 0.05) [40].

### 4.7. Scanning Electron Microscope 

Infected nymphs, winged imagoes, soldiers and workers were fixed by glutaraldehyde (4%) prepared in phosphate buffered-saline (pH 7) with a strength of 0.1 M overnight at 4 °C. After washing thrice in 0.1 M PBS, termite specimens were subsequently fixed for 60 min in 1% Osmium Tetroxide. The fixed specimens were then washed thrice in 0.1 M PBS for subsequent dehydration in different gradients of ethanol (50%, 70%, 80%, 90% and 100%). Drying of all specimens was performed under CO_2_ using a critical-point drying apparatus. After mounting on stubs and coating with gold palladium, specimens were observed at 10 kV under a scanning electron microscope (XL30 ESEM, Philips, Amsterdam, The Netherlands).

### 4.8. Compilation and Analysis of Antioxidant Sequences of Formosan Subterranean Termites

The immunized *C. formosanus* Shiraki cDNA library was constructed by extracting total RNA by TRIzol reagent (Invitrogen, Waltham, MA, USA). The mRNA was isolated from total RNA using the Oligotex mRNA Mini Kit (QIAGEN, Hilden, Germany) by following the supplier’s protocol. After mRNA purification, first-strand cDNA was synthesized by denaturing mRNA and reverse transcription (In-Fusion^TM^ SMARTer^TM^ cDNA library construction kit by Takara Biomedical Technology, Beijing Co., Ltd., Beijing, China). Second-strand cDNA was synthesized by long-distance PCR using a specific primer (5′ PCR Primer II A). The amplified product was purified to normalize the double-strand (ds) cDNAs by the Trimmer-direct cDNA Normalization kit. After purification, the pSMART2IF linearized vector was used to ligate the normalized ds cDNA for subsequent electroporation by competent cells *Escherishia coli* DH5α (Douglas Hanahan 5α). The individual transformants were grown for overnight on LB broth provided with isopropyl-b-d-thiogalactopyranoside (IPTG) and 5-bromo-4-chloro-3-indolyl-b-d-galac-Topyranoside (X-Gal) for sequencing. Expressed sequence tags (ESTs) were annotated using Blast2GO. Antioxidant defense-related ESTs were separately compiled and annotated by BLASTx using NCBI database.

### 4.9. Antioxidant Genes Validation and Quantification by qRT-PCR

First-strand cDNAs reverse transcribed from total RNA extracts of nymphs, winged imagoes, soldiers and workers infected with *M. anisopliae* 02049, *M. anisopliae* 406, *B. bassiana* 03005 and *B. bassiana* 200436 at a concentration of 1.3 × 10^7^ conidia/mL were subjected to qRT-PCR in order to determine the expression patterns of the identified antioxidant genes listed in Table 4. A housekeeping gene, *β-Actin* (forward primer 5′-AGCGGGAAATCGTGCGTGAC-3′ and reverse primer 5′-CAATAGTGATGACCTGGCCGT-3′), was used as an internal control. The specific primers listed in Table 4 were used for quantification using the CFX96 Real-Time System under the conditions mentioned in the our previous study [7]. Three replicates were prepared using three separate termites of each caste. Numerical values obtained from each experimental unit were compared with those of the control by relative fold expression obtained by transforming the obtained results into absolute values using 2^−ΔΔ*C*t^ [41]. In case of control, relative expression of each gene was set to 1. *β-Actin* due to its low expression variations was chosen as housekeeping gene. The relative fold expression pattern of each gene was analyzed by two-factor factorial analysis comprised of four castes and four isolates of entomopathogenic fungi. Significant differences among means were indicated by Fisher’s least significant difference test using SAS version 8 (α = 0.05) [40].

## 5. Conclusions

On the basis of the above findings, we may conclude that the entomopathogenic fungi are only successful in the laboratory because the laboratory evaluation mainly involved the infection of workers, which are not the source of the production of fumigants (naphthalene, *n*-hexanoic acid, nonanal and some other toxic fumigants) in *C. formosanus* Shiraki. Furthermore, the identification of a variety of genes encoding antioxidant defense from *C. formosanus* Shiraki and their upregulation mainly in winged imagoes and workers against the most virulent isolate greatly enhanced the knowledge of the disease resistance mechanism of *C. formosanus* Shiraki. The identified antioxidant transcriptome might lay the initial ground for the recently-developed precise genome editing CRISPR/Cas9 technique that will target all the castes of Formosan subterranean termites.

## Figures and Tables

**Table 1:** Volatile organic compound profiles of *Coptotermes formosanus* Shiraki.

R_t_ (min)	Chemical Formulae	Compounds	RA			
			Winged Imagoes (%)	Nymphs (%)	Workers (%)	Soldiers (%)
8.22	C_6_H_12_O_2_	*n*-Hexanoic acid *	1.35	—	—	—
11.41	C_9_H_18_O	Nonanal *	—	—	—	0.54
12.18	C_9_H_18_O_2_	Methyl octanoate *	—	—	28.17	2.70
13.66	C_10_H_8_	Naphthalene *	20.02	—	—	—
15.95	C_10_H_20_O_2_	*n*-Amyl isovalerate	—	—	37.53	3.39
16.42	C_10_H_12_O_3_	1-Methoxyethyl benzoate	—	16.95	—	—
16.69	C_10_H_12_O	1-Methoxy-4-(1-propenyl)benzene	—	2.36	—	—
16.91	C_10_H_14_O	2-*t*-Butylphenol	—	2.73	—	—
17.05	C_10_H_23_NS_2_	2-Diisopropylaminoethyl ethyl disulfide	—	17.98	—	—
17.30	C_11_H_22_O	*n*-Undecanal	—	—	0.69	0.10
17.61	C_11_H_29_	2,4,6-Trimethyloctane	—	—	1.03	—
17.62	C_12_H_26_	*n*-Dodecane	—	—	—	0.15
17.81	C_12_H_18_O_8_	1,2,3,4-Butanetetrol, tetraacetate, [R *,S *]	—	—	3.27	—
18.24	C_12_H_24_O_3_	2,2-dimethyl-1-(2-hydroxy-1-isopropyl) propyl isobutanoate	—	—	1.03	0.32
19.22	C_14_H_28_O	Tetradecanal	—	—	3.17	0.30
19.43	C_14_H_30_	*n*-Tetradecane *	4.10	—	5.13	0.50
19.55	C_15_H_26_	1,3-Dimethyl-5-*n*-propyl-adamantane	—	2.92	—	—
19.62	C_15_H_24_	β-Caryophyllene	—	1.89	1.17	0.30
19.95	C_15_H_24_	Decahydro-1,1,7[1a,a]-trimethyl-4-methylene-1H-cycloprop[e]azulene	—	—	1.10	0.15
20.42	C_15_H_32_	2,6,10-Trimethyldodecane	4.66	—	3.99	—
20.79	C_15_H_24_O	Butylated hydroxytoluene	2.25	—	—	0.48
20.92	C_15_H_32_	*n*-Pentadecane *	11.29	9.89	—	2.31
21.02	C_15_H_22_	Cadina-1,2,3-triene	—	—	2.58	—
21.80	C_15_H_32_	3-Methyltetradecane	1.28	—	—	—
22.08	C_15_H_26_O	Cedrol	—	6.78	—	—
22.25	C_16_H_34_	*n*-Hexadecane *	15.95	3.95	2.69	0.49
23.02	C_16_H_34_	5-Propyltridecane	12.54	—	—	—
23.20	C_16_H_34_	3-Methylpentadecane	1.66	—	—	—
23.39	C_16_H_18_	1,2,3-Trimethyl-4[E]-propenyl-naphthalene	3.00	34.55	7.23	1.77
23.79	C_17_H_36_	*n*-Heptadecane *	12.54	—	—	—
23.96	C_18_H_38_	2,6,10-Trimethylpentadecane	6.38	—	1.21	—
26.10	C_18_H_38_	*n*-Octadecane	2.97	—	—	—
26.13	C_18_H_32_O_2_	[*Z,Z*]-9,12-Octadecadienoic acid	—	—	—	12.22
26.95	C_18_H_34_O_2_	Oleic acid *	—	—	—	65.84
29.13	C_18_H_36_O_2_	Octadecanoic acid	—	—	—	8.42

R_t_ = retention time; RA = relative area (peak area relative to total peak area); * compounds identified by authentic standard.

**Table 2:** Identified antioxidant defense-related genes of *C. formosanus* Shiraki based on sequence similarity (E ≤ 10^−5^).

No.	Length (bp)	Accession No.	Annotation	Expect Value
1	821	JX876646	*Catalase (CAT)*	5 × 10^−111^
2	957	KC571990	*Dual oxidase 1 (DUOX1)*	7 × 10^−121^
3	847	KC741176	*Glutaredoxin like protein (GRXL)*	1 × 10^−82^
4	920	JX915905	*Glutathione S-transferase (GST)*	2 × 10^−139^
5	1121	JX915906	*Peroxiredoxin (PRXS)*	3 × 10^−161^
6	924	JX915907	*Peroxiredoxin-like protein (PRXSL)*	3 × 10^−105^
7	1237	JX915909	*Peroxiredoxin 1-like protein (PRXS1L)*	5 × 10^−137^
8	852	KC741174	*Superoxide Dismutase Cu/Zn (Cu/Zn–SOD1)*	5 × 10^−103^
9	640	JX311467	*Superoxide Dismutase Fe (Fe–SOD)*	3 × 10^−85^
10	909	JX915904	*Superoxide Dismutase Cu/Zn (Cu/Zn–SOD2)*	4 × 10^−75^
11	892	KC632518	*Thioredoxin family protein (TXN1)*	1 × 10^−58^
12	919	KC741175	*Thioredoxin family protein (TXN2)*	1 × 10^−178^
13	910	JX879125	*Thioredoxin-like protein (TXNL1)*	2 × 10^−51^
14	917	KC741177	*Thioredoxin-like protein (TXNL2)*	5 × 10^−161^
15	849	JX915908	*Thioredoxin-like protein 4A (TXNL4A)*	4 × 10^−60^
16	714	JX879124	*Thioredoxin peroxidase (TPx)*	7 × 10^−88^
17	889	KC632520	*Thioredoxin-like [2Fe–2S] ferredoxin (Fd)* *family protein (TRx-like-Fd)*	5 × 10^−132^

**Table 3:** Expression patterns of *C. formosanus* Shiraki antioxidant genes in the whole body homogenates of winged imagoes, nymphs, soldiers and workers using quantitative real-time PCR (qRT-PCR).

Genes	Castes	Entomopathogenic Fungi				Castes Statistics
		*M. anisopliae* 02049	*M. anisopliae* 406	*B. bassiana* 03005	*B. bassiana* 200436	
*CAT*	Workers	11.15 ± 0.32 ^b^	7.02 ± 0.49 ^d^	5.29 ± 0.26 ^e^	2.98 ± 0.20 ^g^	*F* = 296.74
	Soldiers	2.96 ± 0.28 ^g^	2.12 ± 0.15 ^h^	1.68 ± 0.19 ^hi^	1.13 ± 0.13 ^i^	df = 3, 32
	Nymphs	5.05 ± 0.43 ^e^	3.94 ± 0.19 ^f^	4.92 ± 0.20 ^e^	2.09 ± 0.16 ^h^	*p* < 0.0001
	Winged Imagoes	15.04 ± 0.48 ^a^	3.70 ± 0.35 ^fg^	9.53 ± 0.34 ^c^	1.86 ± 0.16 ^hi^	
	**Infection Statistics**	*F* = 344.94; df = 3, 32; *p* < 0.0001				*F* = 78.83
						df = 9, 32
						*p* < 0.0001
*DUOX1*	Workers	1.07 ± 0.14 ^cde^	0.81 ± 0.07 ^efgh^	1.21 ± 0.11 ^cd^	0.59 ± 0.06 ^hij^	*F* = 24.58
	Soldiers	0.70 ± 0.08 ^ghi^	0.49 ± 0.11 ^ij^	0.98 ± 0.09 ^def^	0.38 ± 0.10 ^j^	df = 3, 32
	Nymphs	1.81 ± 0.12 ^a^	0.89 ± 0.09 ^efg^	1.34 ± 0.11 ^bc^	0.75 ± 0.05 ^fghi^	*p* < 0.0001
	Winged Imagoes	1.59 ± 0.014 ^ab^	0.83 ± 0.08 ^efgh^	1.23 ± 0.08 ^cd^	0.68 ± 0.10 ^ghi^	
	**Infection Statistics**	*F* = 46.44; df = 3, 32; *p* < 0.0001				*F* = 3.36
						df = 9, 32
						*p* = 0.005
*GRXL*	Workers	2.59 ± 0.16 ^d^	1.72 ± 0.12 ^e^	1.82 ± 0.12 ^e^	1.26 ± 0.13 ^fg^	*F* = 238.99
	Soldiers	1.57 ± 0.10 ^ef^	1.06 ± 0.08 ^g^	1.23 ± 0.10 ^fg^	0.91 ± 0.07 ^g^	df = 3, 32
	Nymphs	4.91 ± 0.23 ^a^	1.69 ± 0.10 ^e^	3.77 ± 0.15 ^b^	1.91 ± 0.09 ^e^	*p* < 0.0001
	Winged Imagoes	4.63 ± 0.16 ^a^	3.11 ± 0.10 ^c^	3.93 ± 0.08 ^b^	1.09 ± 0.09 ^g^	
	**Infection Statistics**	*F* = 219.10; df = 3, 32; *p* < 0.0001				*F* = 34.80
						df = 9, 32
						*p* < 0.0001
*GST*	Workers	1.95 ± 0.12 ^ef^	0.91 ± 0.11 ^h^	1.84 ± 0.09 ^ef^	0.67 ± 0.08 ^hi^	*F* = 492.93
	Soldiers	0.88 ± 0.10 ^hi^	0.71 ± 0.08 ^hi^	0.70 ± 0.08 ^hi^	0.61 ± 0.09 ^i^	df = 3, 32
	Nymphs	3.08 ± 0.11 ^c^	2.74 ± 0.12 ^d^	2.11 ± 0.09 ^e^	1.35 ± 0.10 ^g^	*p* < 0.0001
	Winged Imagoes	5.21 ± 0.13 ^a^	1.69 ± 0.10 ^f^	3.94 ± 0.10 ^b^	2.10 ± 0.08 ^e^	
	**Infection Statistics**	*F* = 202.59; df = 3, 32; *p* < 0.0001				*F* = 57.27
						df = 9, 32
						*p* < 0.0001
*PRXS*	Workers	3.88 ± 0.17 ^a^	1.76 ± 0.10 ^e^	2.77 ± 0.18 ^c^	0.83 ± 0.06 ^gh^	*F* = 114.55
	Soldiers	1.84 ± 0.09 ^de^	1.20 ± 0.10 ^f^	1.23 ± 0.11 ^f^	0.76 ± 0.09 ^h^	df = 3, 32
	Nymphs	2.54 ± 0.10 ^c^	1.14 ± 0.09 ^fg^	2.12 ± 0.10 ^d^	0.86 ± 0.09 ^gh^	*p* < 0.0001
	Winged Imagoes	3.73 ± 0.11 ^a^	1.85 ± 0.09 ^de^	3.41 ± 0.11 ^b^	1.10 ± 0.07 ^fg^	
	**Infection Statistics**	*F* = 294.23; df = 3, 32; *p* < 0.0001				*F* = 17.60
						df = 9, 32
						*p* < 0.0001
*PRXSL*	Workers	2.47 ± 0.12 ^b^	2.05 ± 0.08 ^cd^	1.90 ± 0.10 ^d^	0.96 ± 0.10 ^ef^	*F* = 65.96
	Soldiers	1.85 ± 0.09 ^d^	1.23 ± 0.10 ^e^	1.07 ± 0.08 ^ef^	0.77 ± 0.06 ^f^	df = 3, 32
	Nymphs	2.06 ± 0.10 ^cd^	1.84 ± 0.11 ^d^	1.17 ± 0.10 ^e^	0.80 ± 0.08 ^f^	*p* < 0.0001
	Winged Imagoes	3.49 ± 0.16 ^a^	1.77 ± 0.11 ^d^	2.28 ± 0.13 ^bc^	1.18 ± 0.08 ^e^	
	**Infection Statistics**	*F* = 149.75; df = 3, 32; *p* < 0.0001				*F* = 10.27
						df = 9, 32
						*p* < 0.0001
*PRXS1L*	Workers	1.01 ± 0.09 ^cdef^	0.79 ± 0.08 ^fg^	1.07 ± 0.09 ^bcde^	0.48 ± 0.09 ^h^	*F* = 13.70
	Soldiers	1.86 ± 0.10 ^a^	1.03 ± 0.09 ^cdef^	0.96 ± 0.08 ^def^	0.65 ± 0.08 ^gh^	df = 3, 32
	Nymphs	0.96 ± 0.10 ^def^	0.86 ± 0.08 ^efg^	1.11 ± 0.08 ^bcd^	1.22 ± 0.09 ^bc^	*p* < 0.0001
	Winged Imagoes	1.29 ± 0.10 ^b^	1.19 ± 0.07 ^bcd^	1.14 ± 0.07 ^bcd^	1.17 ± 0.08 ^bcd^	
	**Infection Statistics**	*F* = 16.54; df = 3, 32; *p* < 0.0001				*F* = 11.66
						df = 9, 32
						*p* < 0.0001
*Cu/Zn–SOD1*	Workers	1.10 ± 0.08 ^fgh^	1.24 ± 0.08 ^ef^	1.05 ± 0.07 ^fgh^	0.84 ± 0.07 ^h^	*F* = 325.13
	Soldiers	1.09 ± 0.08 ^fgh^	0.88 ± 0.09 ^gh^	1.21 ± 0.09 ^ef^	1.10 ± 0.08 ^fgh^	df = 3, 32
	Nymphs	3.74 ± 0.12 ^a^	1.44 ± 0.09 ^e^	2.89 ± 0.12 ^c^	1.89 ± 0.09 ^d^	*p* < 0.0001
	Winged Imagoes	3.17 ± 0.10 ^b^	2.73 ± 0.10 ^c^	2.91 ± 0.12 ^bc^	1.10 ± 0.06 ^fg^	
	**Infection Statistics**	*F* = 103.32; df = 3, 32; *p* < 0.0001				*F* = 45.14
						df = 9, 32
						*p* < 0.0001
*Fe–SOD*	Workers	3.88 ± 0.15 ^b^	3.16 ± 0.13 ^d^	3.67 ± 0.11 ^bc^	2.66 ± 0.10 ^e^	*F* = 344.02
	Soldiers	1.23 ± 0.08 ^g^	1.11 ± 0.08 ^g^	0.92 ± 0.08 ^gh^	0.62 ± 0.11 ^h^	df = 3, 32
	Nymphs	4.21 ± 0.12 ^a^	3.69 ± 0.12 ^bc^	2.14 ± 0.10 ^f^	1.11 ± 0.07 ^g^	*p* < 0.0001
	Winged Imagoes	3.51 ± 0.13 ^c^	2.24 ± 0.12 ^f^	2.94 ± 0.13 ^de^	2.12 ± 0.10 ^f^	
	**Infection Statistics**	*F* = 137.33; df = 3, 32; *p* < 0.0001				*F* = 30.98
						df = 9, 32
						*p* < 0.0001
*Cu/Zn–SOD2*	Workers	4.35 ± 0.19 ^b^	2.96 ± 0.10 ^c^	1.05 ± 0.09 ^efg^	0.77 ± 0.09 ^gh^	*F* = 248.02
	Soldiers	1.02 ± 0.09 ^efg^	1.21 ± 0.08 ^e^	0.84 ± 0.09 ^fgh^	0.52 ± 0.08 ^h^	df = 3, 32
	Nymphs	2.88 ± 0.12 ^c^	1.93 ± 0.14 ^d^	1.66 ± 0.11 ^d^	1.12 ± 0.07 ^ef^	*p* < 0.0001
	Winged Imagoes	5.99 ± 0.16 ^a^	3.13 ± 0.12 ^c^	1.12 ± 0.08 ^ef^	1.92 ± 0.12 ^d^	
	**Infection Statistics**	*F* = 422.72; df = 3, 32; *p* < 0.0001				*F* = 68.00
						df = 9, 32
						*p* < 0.0001
*TXN1*	Workers	2.74 ± 0.12 ^ab^	2.06 ± 0.11 ^de^	1.73 ± 0.12 ^fg^	1.13 ± 0.08 ^i^	*F* = 32.14
	Soldiers	1.82 ± 0.17 ^efg^	1.55 ± 0.12 ^gh^	1.12 ± 0.09 ^ij^	0.81 ± 0.07 ^j^	df = 3, 32
	Nymphs	2.47 ± 0.10 ^bc^	1.92 ± 0.10 ^def^	2.18 ± 0.12 ^cd^	1.27 ± 0.10 ^hi^	*p* < 0.0001
	Winged Imagoes	2.96 ± 0.11 ^a^	1.76 ± 0.10 ^efg^	1.94 ± 0.12 ^def^	1.14 ± 0.07 ^i^	
	**Infection Statistics**	*F* = 111.59; df = 3, 32; *p* < 0.0001				*F* = 4.34
						df = 9, 32
						*p* < 0.0001
*TXN2*	Workers	2.13 ± 0.11 ^c^	1.89 ± 0.13 ^cde^	0.87 ± 0.10 ^g^	0.86 ± 0.09 ^g^	*F* = 24.49
	Soldiers	1.94 ± 0.11 ^cd^	1.48 ± 0.11 ^f^	1.13 ± 0.06 ^g^	0.93 ± 0.11 ^g^	df = 3, 32
	Nymphs	2.76 ± 0.13 ^b^	1.73 ± 0.13 ^def^	1.91 ± 0.12 ^cde^	1.09 ± 0.08 ^g^	*p* < 0.0001
	Winged Imagoes	3.75 ± 0.14 ^a^	1.17 ± 0.06 ^g^	1.60 ± 0.11 ^ef^	0.92 ± 0.12 ^g^	
	**Infection Statistics**	*F* = 176.34; df = 3, 32; *p* < 0.0001				*F* = 19.92
						df = 9, 32
						*p* = 0.0009
*TXNL1*	Workers	1.76 ± 0.14 ^ab^	0.81 ± 0.09 ^e^	1.38 ± 0.11 ^cd^	0.21 ± 0.05 ^g^	*F* = 0.46
	Soldiers	1.91 ± 0.11 ^a^	0.65 ± 0.09 ^ef^	1.17 ± 0.12 ^d^	0.47 ± 0.09 ^fg^	df = 3, 32
	Nymphs	1.50 ± 0.10 ^bc^	1.16 ± 0.07 ^d^	1.29 ± 0.11 ^cd^	0.48 ± 0.08 ^fg^	*p* > 0.05
	Winged Imagoes	1.93 ± 0.12 ^a^	1.15 ± 0.11 ^d^	0.84 ± 0.08 ^e^	0.26 ± 0.08 ^g^	
	**Infection statistics**	*F* = 142.88; df = 3, 32; *p* < 0.0001				*F* = 6.06
						df = 9, 32
						*p* < 0.0001
*TXNL2*	Workers	2.11 ± 0.11 ^b^	1.18 ± 0.11 ^de^	1.86 ± 0.11 ^bc^	0.68 ± 0.09 ^f^	*F* = 15.95
	Soldiers	1.96 ± 0.14 ^bc^	1.13 ± 0.06 ^de^	1.72 ± 0.11 ^c^	0.79 ± 0.09 ^f^	df = 3, 32
	Nymphs	2.17 ± 0.13 ^b^	1.67 ± 0.12 ^c^	0.93 ± 0.07 ^ef^	0.65 ± 0.12 ^f^	*p* < 0.0001
	Winged Imagoes	2.79 ± 0.15 ^a^	1.16 ± 0.07 ^de^	2.17 ± 0.11 ^b^	1.26 ± 0.14 ^d^	
	**Infection Statistics**	*F* = 115.31; df = 3, 32; *p* < 0.0001				*F* = 9.56
						df = 9, 32
						*p* < 0.0001
*TXNL4A*	Workers	4.73 ± 0.15 ^b^	2.82 ± 0.14 ^ef^	3.46 ± 0.16 ^d^	1.13 ± 0.08 ^j^	*F* = 159.16
	Soldiers	2.09 ± 0.14 ^gh^	1.53 ± 0.12 ^i^	1.91 ± 0.14 ^hi^	1.07 ± 0.07 ^j^	df = 3, 32
	Nymphs	3.86 ± 0.14 ^c^	2.30 ± 0.12 ^g^	3.20 ± 0.13 ^de^	1.94 ± 0.13 ^gh^	*p* < 0.0001
	Winged Imagoes	5.31 ± 0.18 ^a^	2.72 ± 0.13 ^f^	4.76 ± 0.16 ^b^	2.13 ± 0.12 ^gh^	
	**Infection Statistics**	*F* = 253.32; df = 3, 32; *p* < 0.0001				*F* = 20.22
						df = 9, 32
						*p* < 0.0001
*TPx*	Workers	3.74 ± 0.15 ^ab^	2.46 ± 0.10 ^g^	3.35 ± 0.15 ^cd^	1.73 ± 0.12 ^h^	*F* = 142.07
	Soldiers	1.88 ± 0.13 ^h^	1.12 ± 0.09 ^i^	1.21 ± 0.10 ^i^	0.68 ± 0.12 ^j^	df = 3, 32
	Nymphs	3.47 ± 0.16 ^bc^	1.94 ± 0.13 ^h^	2.69 ± 0.14 ^gh^	1.12 ± 0.08 ^i^	*p* < 0.0001
	Winged Imagoes	3.95 ± 0.14 ^a^	3.09 ± 0.12 ^de^	2.93 ± 0.14 ^ef^	1.08 ± 0.08 ^i^	
	**Infection Statistics**	*F* = 201.29; df = 3, 32; *p* < 0.0001				*F* = 9.66
						df = 9, 32
						*p* < 0.0001
*TRx-like-Fd*	Workers	3.53 ± 0.16 ^a^	2.82 ± 0.17 ^b^	3.05 ± 0.14 ^b^	1.65 ± 0.12 ^d^	*F* = 84.27
	Soldiers	1.82 ± 0.15 ^cd^	1.26 ± 0.13 ^e^	1.12 ± 0.07 ^ef^	0.73 ± 0.09 ^g^	df = 3, 32
	Nymphs	2.79 ± 0.16 ^b^	2.14 ± 0.10 ^c^	1.68 ± 0.13 ^d^	0.76 ± 0.09 ^fg^	*p* < 0.0001
	Winged Imagoes	3.07 ± 0.11 ^b^	2.18 ± 0.14 ^c^	2.19 ± 0.12 ^c^	1.25 ± 0.13 ^e^	
	**Infection Statistics**	*F* = 117.62; df = 3, 32; *p* < 0.0001				*F* = 7.84
						df = 9, 32
						*p* < 0.0001

Relative fold expression values are the means of three replicates. Significant differences among the means were analyzed by two-factor factorial ANOVA under completely randomized design (CRD) (Fisher’s LSD test, α = 0.05). Means ± SE values with the same letter(s) are not significantly different.

**Table 4:** Antioxidant genes used to study expression patterns of the whole body homogenates of winged imagoes, nymphs, soldiers and workers of *C. formosanus* Shiraki using quantitative real-time PCR (qRT-PCR).

Gene	Product Length	Accession Number	Forward Primer (5′-3′)	Reverse Primer (5′-3′)
*Catalase (CAT)*	97 bp	JX876646	TGGCACCAACTACCTTCAAA	TCCTTGGTGATGCATTGTTT
*Dual oxidase 1 (DUOX1)*	95 bp	KC571990	AGTTTGACCTCAGGACCATCC	AGTGACTGCTTTCAGCCCAG
*Glutaredoxin like protein (GRXL)*	87 bp	KC741176	GGAATTGCATCCAGTAGGGAGA	CTGCTCCCTTGCCTCTTCTT
*Glutathione S-transferase (GST)*	99 bp	JX915905	GTATTGGCAGGTTCGTGTTG	TACAGCCTCAGCTTCCCTTT
*Peroxiredoxin (PRXS)*	106 bp	JX915906	ACCTGTTGGTCGCAGTGTAG	TTCTTGTCCTGGCTTCCAGC
*Peroxiredoxin-like protein (PRXSL)*	112 bp	JX915907	TTCATGCTGTAGGGCGTGTT	TCACAGAGCTCCACACTTGG
*Peroxiredoxin 1-like protein (PRXS1L)*	97 bp	JX915909	GCATATTCTGACCGTGCAGC	TGTTGACCCAAGCAAGGTGA
*Superoxide Dismutase Cu/Zn (Cu/Zn-SOD1)*	93 bp	KC741174	GTAACGGGGGAAGTGACTGG	TCCAGCACTTGTACAGCCAT
*Superoxide Dismutase Fe (Fe-SOD)*	127 bp	JX311467	ACAGTTGATGCTTGGGAACA	TGAGACCAGCAGCCTTAAAC
*Superoxide Dismutase Cu/Zn (Cu/Zn-SOD2)*	90 bp	JX915904	AATGGAGAAGTGGTCAAGGG	TGACAGACCAGTCACTTCCC
*Thioredoxin family protein (TXN1)*	81 bp	KC632518	CTTACACCGGAGGACGACAT	AACCATCGGGTGCTCGATTC
*Thioredoxin family protein (TXN2)*	75 bp	KC741175	TCTGAATGTTGCCCGTGTGA	GGCACTTCCCAGACTCCAAA
*Thioredoxin-like protein (TXNL1)*	97 bp	JX879125	ACAAAGCTTACGGAGGCAGG	GCTCCTCAATTCTGGGTGCT
*Thioredoxin-like protein (TXNL2)*	81 bp	KC741177	GGCCACACCACAACTAAAGC	ATGCAGGTGTTTTGGTCGGA
*Thioredoxin-like protein 4A (TXNL4A)*	80 bp	JX915908	TGGCCATTGGAAGACAAGCA	ACTAGACCTCGACCTTTGCG
*Thioredoxin peroxidase (TPx)*	80 bp	JX879124	TGCCGCAAAACATGGAGAAG	TGCTCTCATTCGGCTTAGGA
*Thioredoxin-like [2Fe–2S] ferredoxin (Fd) family protein (TRx-like-Fd)*	106 bp	KC632520	CTTGTGTCAACGCCCCAATG	GGCACTCGGCCATTTTTCAA

## Abbreviations

HS-SPME	Head space-solid phase micro-extraction
VOCs	Volatile organic compounds
dp aps	Directly-penetrating appressorium-like structures
co	Unipolar-germinated conidium
CAT	Catalase
DUOX1	Dual oxidase 1
GRXL	Glutaredoxin-like protein
GST	Glutathione S-transferase
PRXS	Peroxiredoxin
PRXSL	Peroxiredoxin-like protein
PRXS1L	Peroxiredoxin 1-like protein
Cu/Zn-SOD	Superoxide dismutase Cu/Zn
Fe-SOD	Superoxide dismutase Fe
TXN	Thioredoxin family protein
TXNL	Thioredoxin-like protein
TPx	Thioredoxin peroxidase
TRx-like-Fd	Thioredoxin-like [2Fe–2S] ferredoxin (Fd) family protein
SEM	Scanning electron microscope
ESTs	Expressed sequence tags
